# Correlation of dynamic contrast-enhanced MRI and diffusion-weighted MR imaging with prognostic factors and subtypes of breast cancers

**DOI:** 10.3389/fonc.2022.942943

**Published:** 2022-08-05

**Authors:** Hui Chen, Wei Li, Chao Wan, Jue Zhang

**Affiliations:** ^1^ Department of Oncology, Tianmen First People’s Hospital, Tianmen, China; ^2^ Department of CT/MRI, Tianmen First People's Hospital, Tianmen, China

**Keywords:** ADC value, dynamic contrast-enhanced MR imaging, diffusion-weighted MR imaging, breast cancer, molecular subtypes

## Abstract

**Objective:**

To determine the preoperative magnetic resonance imaging (MRI) findings of breast cancer on dynamic contrast-enhanced magnetic resonance imaging (DCE-MRI) and diffusion-weighted magnetic resonance imaging (DWI) in different molecular subtypes.

**Materials and methods:**

A retrospective study was conducted on 116 breast cancer subjects who underwent preoperative MRI and surgery or biopsy. Three radiologists retrospectively assessed the morphological and kinetic characteristics on DCE-MRI and tumor detectability on DWI, by using apparent diffusion coefficient (ADC) values of lesions. The clinicopathologic and MRI features of four subtypes were compared. The correlation between clinical and MRI findings with molecular subtypes was evaluated using the chi-square and ANOVA tests, while the Mann–Whitney test was used to analyze the relationship between ADC and prognostic factors.

**Results:**

One hundred and sixteen women diagnosed with breast cancer confirmed by surgery or biopsy had the following subtypes of breast cancer: luminal A (27, 23.3%), luminal B (56, 48.2%), HER2 positive (14, 12.1%), and triple-negative breast cancer (TNBC) (19, 16.4%), respectively. Among the subtypes, significant differences were found in axillary node metastasis, histological grade, tumor shape, rim enhancement, margin, lesion type, intratumoral T2 signal intensity, Ki-67 index, and paratumoral enhancement (*p* < 0.001, *p* < 0.001, *p* < 0.001, *p* < 0.001, *p* < 0.001, *p* < 0.001, *p* < 0.001, *p* < 0.001, and *p* = 0.02, respectively). On DWI, the mean ADC value of TNBC (0.910 × 10^−3^ mm^2^/s) was the lowest compared to luminal A (1.477×10^−3^ mm^2^/s), luminal B (0.955 × 10^−3^ mm^2^/s), and HER2 positive (0.996 × 10^−3^ mm^2^/s) (*p* < 0.001). Analysis of the correlation between different prognostic factors and ADC value showed that only axillary lymph node status and ADC value had a statistically significant difference (*p* = 0.009).

**Conclusion:**

The morphologic features of MRI can be used as imaging biomarkers to identify the molecular subtypes of breast cancer. In addition, quantitative assessments of ADC values on DWI may also provide biological clues about molecular subtypes.

## Introduction

Breast cancer is a group of heterogeneous diseases with different molecular subtypes, morphological features, clinical behaviors, and treatment responses. For a better patient-based approach, one of the most important indicators to evaluate disease and its prognosis is the molecular subtype, together with tumor size, histological grade, and the presence of metastatic axillary lymph nodes (1, 2). In addition to these, other standard histological factors are useful to determine different prognoses and management of the disease, including histological grade, the Ki-67 proliferation index, and the expression of the estrogen receptor (ER), the progesterone receptor (PR), and the human epidermal growth factor receptor 2 (HER2) (3). By immunohistochemistry and fluorescence *in-situ* hybridization, the current commonly accepted molecular subtypes include luminal A (ER+/PR+/HER2−, Ki-67 < 15%), luminal B (ER+/PR+ or –/HER2 positive or negative, Ki-67 ≥ 15%), HER2-enriched (EP−/PR−, HER2 positive), and triple-negative breast cancer (TNBC) (ER−/RP−, HER2 negative). Several studies have confirmed that distinct molecular subtypes respond differently to therapy and are related to different prognoses: luminal A is usually the most common molecular subtype and typically confers the best prognosis, luminal B shows a good response to radiation therapy and has intermediate survival, and HER2-enriched and triple-negative breast cancer have a good response to chemotherapy but the worst overall survival (4, 5).

Dynamic contrast-enhanced magnetic resonance imaging (DCE-MRI) is the most accurate and the highest sensitivity diagnostic imaging technique for detecting breast cancer, which might not be identified with mammography or ultrasound (6, 7). In the case of breast cancer, the ability to predict tumor molecular subtypes with imaging may provide an important contribution to clinical practice of early treatment planning and understanding of prognosis. Until now, very little is known about the diffusion-weighted MRI (DWI) characteristics of different subtypes of breast cancer (8). By studying the underlying biological and functional characteristics, DWI is expected to eventually improve our understanding of the subtypes of breast cancer, especially prognosis and treatment plans (9–13). The aim of our study was to investigate the MRI features of the molecular subtypes of cancer in patients using DCE-MRI and DWI.

## Materials and methods

### Patient selection

The local institutional review board approved this retrospective study, and the informed consent requirement was waived. A retrospective analysis was performed on 116 women aged 26–74 years who underwent breast magnetic resonance examination and have been submitted to biopsy or surgery with the diagnosis of breast cancer in our hospital from September 2017 to March 2022. The following exclusion criteria were applied: 1) patients treated with neoadjuvant chemotherapy; 2) patients with incomplete information on ER, PR, and HER2 status; and 3) those who dropped treatment or did not receive follow-up treatment in our hospital.

### Histopathologic assessment

Serial slices of specimens from breast-conserving surgery or from mastectomy were analyzed by one pathologist who evaluated the size of the tumor, axillary node invasion, and histopathologic grade according to the Elston–Ellis classification and then classified the histotype according to the World Health Organization system. The tissue specimens were fixed with 10% formaldehyde, embedded in paraffin, sliced into 5-μm-thick sections, and stained with hematoxylin and eosin (HE). The receptor status was considered positive if the expression of each receptor was 10% or greater. In HER2 immunohistochemical staining, a score of 0 or 1+ was negative, 3+ was positive, and 2+ was equivocal, and the status of patients was verified using fluorescence *in-situ* hybridization (FISH), where FISH results were either positive or negative. Breast cancer was classified into four types according to the expression of ER/PR/HER2 in immunohistochemistry.

### Imaging protocol

All breast MR examinations were performed using a 3.0-T MRI system (Signa Pioneer, GE Healthcare (Boston, USA)) in a prone position using dedicated bilateral breast surface coils. Each study included a precontrast non-fat-saturated T1-weighted sequence, a precontrast fat-saturated T2-weighted sequence, and DWI (with two *b*-values, 0 and 1,000 s/mm^2^). Gadolinium with meglumine Magenwijan (Guangzhou, China) was administered intravenously at 0.2 mmol/kg. The images were collected once before the contrast scan with 3D Vibrant technology (California, USA), and then eight images within 6 min should be collected after contrast injection. All the 3D Vibrant images used the ReadyView dynamic enhancement curve post-processing.

### Image interpretation

Magnetic resonance imaging including DWI was independently reviewed by three radiologists (with 15, 9, and 6 years of experience in breast MRI, respectively), using the American College of Radiology BI-RADS (Breast Imaging Reporting and Data System) MR lexicon (14). All of them were blinded to clinical and pathologic information. The conclusions of the three radiologists were compared and discordances were resolved by consensus. The MR imaging findings were evaluated for lymph node involvement, morphological characteristics (margin, shape, T2 intensity), rim enhancement, and contrast enhancement kinetics, while kinetic analysis was evaluated with a time–intensity curve (TIC). TIC is based on a region of interest (ROI) that is plotted on the brightest enhancement region to avoid bleeding and necrosis. In the end, morphological manifestations, enhancement types, and TIC types of lesions were analyzed and recorded.

### Statistical analysis

The chi-square test or Fisher’s exact test was used to compare the clinicopathological features among the four tumor subtypes for categorical variables and the ANOVA test for continuous variables. Categorical data were presented as frequency and percentage, whereas continuous data were presented as mean and standard deviation. To evaluate the normality of the quantitative variable distributions, the Mann–Whitney test and the Kruskal–Wallis test were carried out. All analyses were performed using SPSS version 23 (SPSS Inc., SPSS^®^, Chicago, IL, USA), with *p <*0.05 considered to indicate a significant difference.

## Results

### Clinicopathological features

The clinicopathological features of the patients are summarized in **Table 1**. Of the 116 invasive breast cancers, 27 (23.3%) were classified as luminal A, 56 (48.2%) as luminal B, 14 (12.1%) as HER2-enriched, and 19 (16.4%) as TNBC. The mean age of the patients was 51.90 ± 10.68 years (range 26 to 74). In our study, invasive ductal carcinoma was the main pathologic type (107 cases, 92.3%), and there were 9 cases only (accounting for 7.7%) of invasive lobular carcinoma and other types of breast cancer. The highest histological grade (grade 3) was associated with HER2-enriched and TNBC compared to the luminal subtypes. Tumor histological grade was significantly different among the four subtypes (*p* < 0.001), as well as the mean Ki-67 index (*p* < 0.001) and the presence of axillary nodal status (*p* < 0.001). However, there were no differences in age and tumor sizes.

**Table 1:** Clinicopathological features stratified by molecular subtypes.

Tumor subtype	Luminal A	Luminal B	HER2-enriched	TNBC	*p*-value
Patient age (years)	50.74 ± 11.19	52.33 ± 10.92	51.07 ± 6.70	52.84 ± 12.13	0.889
Ki-67 index	7.78 ± 2.53	46.88 ± 21.52	48.93 ± 19.13	53.42 ± 25.44	<0.001
Histological grade					<0.001
Grade 1	17 (63%)	3 (5.3%)	1 (7.1%)	2 (10.5%)	
Grade 2	9 (33.3%)	30 (53.6%)	4 (28.6%)	3 (15.8%)	
Grade 3	1 (3.7%)	23 (41.1%)	9 (64.3%)	14 (73.7%)	
Axillary lymph node					<0.001
Positive	5 (18.5%)	36 (64.3%)	11 (78.6%)	10 (52.6%)	
Negative	22 (81.5%)	20 (35.7%)	3 (21.4%)	9 (47.4%)	

### MR imaging features

**Figure 1:**
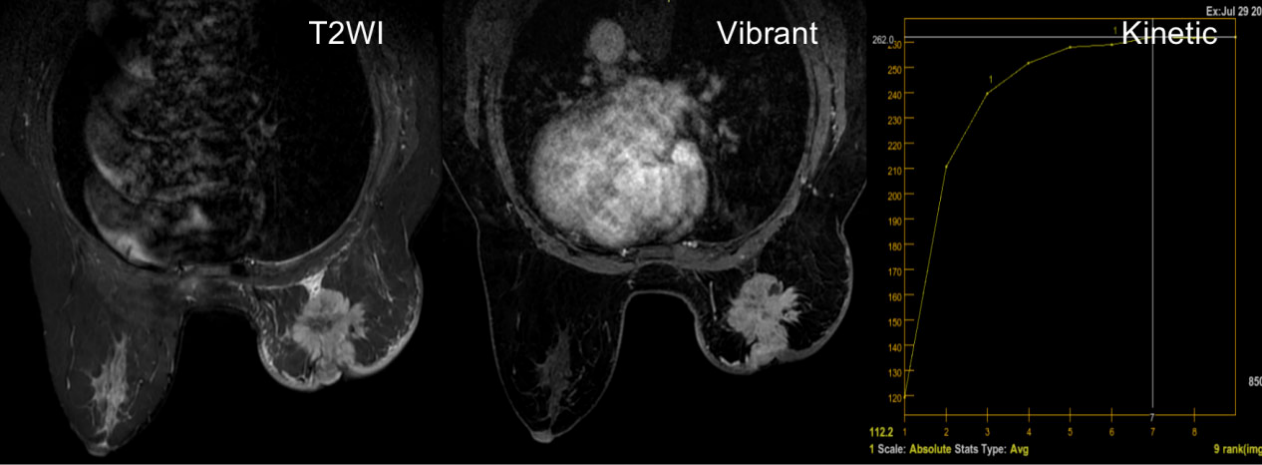
A 53-year-old woman diagnosed with invasive ductal carcinoma of the luminal B subtype. The T2-weighted image shows a strong hyperintense signal inside the mass without enhancement on subtracted images, representing necrosis. The Vibrant technology shows an irregular mass with an irregular margin and a heterogeneous enhancement. Kinetic curves generated from two regions of the enhanced ring demonstrate a plateau appearance (type II curve).

**Figure 2:**
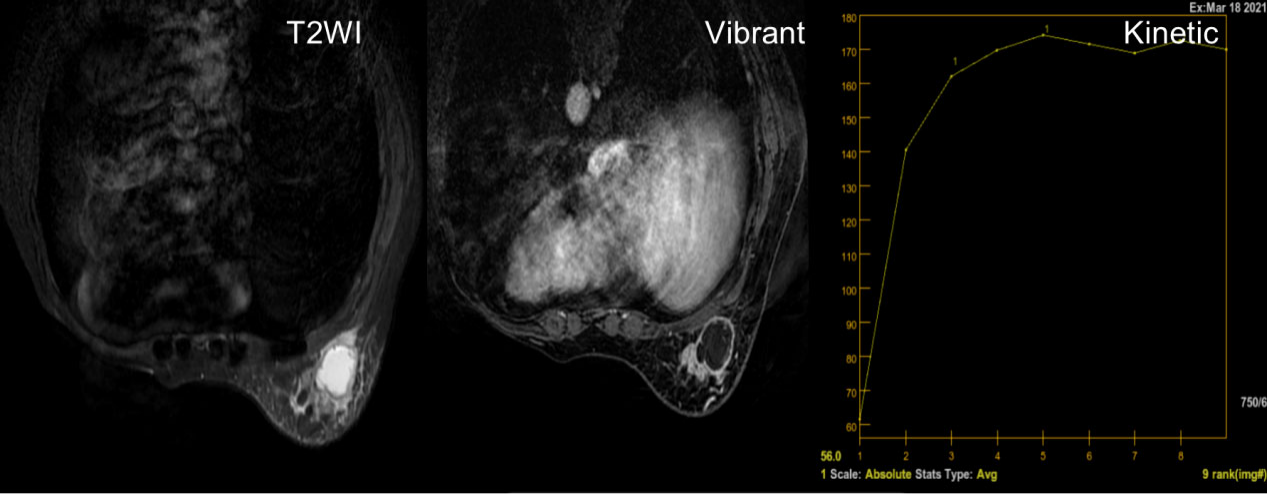
A 50-year-old woman with a solitary hyperintensity in T2 lesion, with rim enhancement in Vibrant and a type II kinetic curve (plateau).

In our study, mass lesions were the most commonly detected in MRI (94%). MR imaging features stratified by molecular subtypes are summarized in **Table 2** and two cases are shown in **Figures 1**, **Figure 2**. On DCE-MRI, the differences in tumor shape, internal enhancement mode, tumor margin, tumor type, and intratumoral T2 signal intensity among the molecular types were statistically significant between groups (*p* < 0.001, *p* < 0.001, *p* < 0.001, *p* < 0.001, respectively). Compared to other molecular types, TNBC was more likely to present a regular shape (73.7%), rim enhancement (73.7%), unifocal tumor (78.9%), smooth margin (89.5%), and higher intratumor enhancement of T2 by Bonferroni-adjusted multiple comparisons (89.5%). Moreover, we found that all TNBC patients presented with medium/high T2 signal. Although TNBC in the study was more frequently detected as unifocal lesions than other subtypes (78.9%), the difference was not statistically significant. A detailed analysis of the kinetic curves has shown that all cases have a similar behavior, reaching a plateau before washing out. After dividing the cases into three groups with respect to the tumor diameter (<2, ≥2, <5, ≥5 cm), it was found that there was no significant difference in the distribution of each curve among subgroups as well as tumor size. In addition, the comparative analysis of paratumor signal intensity showed statistically significant differences among subtypes (*p* = 0.02), which could be better used for molecular typing identification.

**Table 2:** MR imaging features stratified by molecular subtypes.

Tumor subtype(case)	Luminal A(27)	Luminal B(56)	HER2-enriched(14)	TNBC(19)	P value
Shape					<0.001
Regular	6 (22.2%)	9 (16.1%)	5 (35.7%)	14 (73.7%)	
Irregular	21 (77.8%)	47 (83.9%)	9 (64.3%)	5 (26.3%)	
Internal **enhancement**					<0.001
Rim	5 (18.5%)	36 (64.3%)	11 (78.6%)	14 (73.7%)	
Heterogeneous	22 (81.5%)	20 (35.7%)	3 (21.4%)	5 (26.3%)	
Tumor **number**					0.284
Unifocal	8 (29.6%)	24 (42.9%)	5 (35.7%)	15 (78.9%)	
Multifocal	19 (70.4%)	32 (57.1%)	9 (64.3%)	4 (21.1%)	
Margin					<0.001
Smooth	7 (25.9%)	7 (12.5%)	3 (21.4%)	17 (89.5%)	
Irregular	20 (74.1%)	49 (87.5%)	11 (78.6%)	2 (10.5%)	
Lesion **type**					<0.001
Mass	24 (88.9%)	52 (92.9%)	14 (100%)	19 (100%)	
Non-mass	3 (11.1%)	4 (7.1%)	0 (0)	0 (0)	
Kinetic **curve pattern**					0.46
Persistent	0 (0)	0 (0)	0 (0)	0 (0)	
Plateau	12 (44.4%)	17 (30.3%)	5 (35.7%)	9 (47.4%)	
Washout	15 (55.6%)	39 (69.7%)	9 (64.3%)	10 (52.6%)	
Tumor size (cm)					0.755
<2	7 (25.9%)	12 (21.4%)	3 (21.4%)	6 (31.6%)	
≥2,<5	17 (63.0%)	31 (55.4%)	8 (57.2%)	8 (42.1%)	
≥5	3 (11.1%)	13 (23.2%)	3 (21.4%)	5 (26.3%)	
Paratumoral enhancement					0.02
Yes	10 (37.0%)	40 (71.4%)	10 (71.4%)	13 (68.4%)	
No	17 (63.0%)	16 (28.6%)	4 (28.6%)	6 (31.6%)	
Intratumoral SI on T2WI					<0.001
Low	4 (14.8%)	7 (12.5%)	3 (21.4%)	0 (0)	
Equal	19 (70.4%)	45 (80.4%)	10 (71.4%)	2 (10.5%)	
High/Very high	4 (14.8%)	4 (7.1%)	1 (7.2%)	17 (89.5%)	
ADC value (×10^−3^ mm^2^/s)	1.477 ± 0.380	0.955 ± 0.190	0.996 ± 0.116	0.910 ± 0.184	<0.001
	(0.649-2.204)	(0.575-1.464)	(0.830-1.262)	(0.654-1.347)	

### Correlation between the prognostic factors and apparent diffusion coefficient values

On DWI, the mean apparent diffusion coefficient (ADC) value of TNBC (0.910 × 10^−3^ mm^2^/s) was lower compared to the mean ADC values for luminal A, luminal B, and HER2+ (1.477, 0.955, and 0.996; *p* < 0.001) (**Table 2**). The correlation between the prognostic factors and ADC values is summarized in **Table 3**. The average ADC values of the ER-positive (84, 72.4%) and PR-positive (72, 62.1%) groups were greater than those of the ER- and PR-negative ones (0.993 × 10^−3^ vs. 0.941 × 10^−3^ mm^2^/s, 1.002 × 10^−3^ vs. 0.940 × 10^−3^ mm^2^/s). However, the difference between the ADC values of the HER2 and axillary lymph nodes under different states was higher in the negative group (1.001 × 10^−3^ vs. 0.923 × 10^−3^ mm^2^/s, 1.078 × 10^−3^ vs. 0.892 × 10^−3^ mm^2^/s). The difference was statistically significant only in axillary lymph node status (*p* = 0.009).

**Table 3:** The correlation between the prognostic factors and ADC values.

Prognostic factors	Case	ADC value (×10^−3^ mm^2^/s)	*p*-value
ER			0.956
Positive	84 (72.4%)	0.993 ± 0.352 (0.429–2.204)	
Negative	32 (27.6%)	0.941 ± 0.179 (0.654–1.347)	
PR			0.959
Positive	72 (62.1%)	1.002 ± 0.377 (0.429–2.204)	
Negative	44 (37.9%)	0.940 ± 0.162 (0.654–1.347)	
HER2			0.553
Positive	33 (28.4%)	0.923 ± 0.171 (0.575–1.283)	
Negative	83 (71.6%)	1.001 ± 0.354 (0.429–2.204)	
Axillary lymph node			0.009
Positive	62 (53.4%)	0.892 ± 0.209 (0.429–1.361)	
Negative	54 (46.6%)	1.078 ± 0.381 (0.456–2.204)	

## Discussion

Knowing the molecular subtypes of breast cancer is key to defining a correct, patient-oriented plan. The different molecular subtypes of breast cancer could have different initial symptoms and metastatic spread and respond differently to radiotherapy and chemotherapy (15). These findings suggest that diagnostic tests, treatment strategies, and surveillance may better guide the collection of information from each patient’s specific molecular subtype of breast cancer. Our study provides an additional step in that direction by identifying clinical findings between different molecular subtypes, which may help guide the preoperative use of breast MR imaging.

The cancer subtype has been shown to be a key condition to determine the correct treatment. As of today, though, the existence of axillary lymph node metastasis still determines the treatment sequence (preoperative vs. postoperative), the type of therapy (endocrine, chemotherapy, and/or targeted therapy), and the drugs and cycle used (16, 17). Lymph node status is also helpful to estimate the prognosis and the consequent benefits of systemic therapies. The clinical approaches to the assessment and treatment of axillary breast cancer in the early stage are evolving and are guided by studies supporting less aggressive surgery (18) and more aggressive radiotherapy for lymph node-positive disease (19). However, the relationship between tumor subtypes and axillary lymph node status is currently unclear. Previous studies have had conflicting results on the incidence of lymph node metastasis in TNBC, with no clear evidence of increase in axillary lymph node metastasis in more aggressive tumors (20, 21). Our study found that pathological analysis confirmed the presence of metastatic lymph node metastasis in luminal B and HER2-positive breast cancer, which was consistent with Grimm et al. Because HER2 and luminal B subtypes are easier to diagnose, the clinical use of MRI to help guide treatment plans such as axillary management and systemic therapy may be more effective for HER2 and luminal B subtypes and may influence clinical outcomes (22).

Breast cancer subtypes have some specific imaging features. From the literature, we know that some particular characteristics of TNBC can be found on breast MRI, such as regular shape, smooth edge, rim enhancement, unifocal lesion, higher histological grade, and high intratumoral signal intensity on T2-weighted images (23–27). In contrast, the luminal type of breast cancer showed more irregular-shaped masses on MRI (24, 27), which was consistent with our findings. In our study, 73.7% of TNBC showed rim enhancement. Navarro Vilar et al. (27) confirmed that 68.7% of TNBC tumors had rim enhancement. Based on this conclusion, the authors pointed out that rim enhancement of the mass is the most useful finding for predicting TNBC. According to relevant literature, the incidence of rim enhancement in TNBC varies from 41% to 80% (7, 24, 27), and our findings are also within this range. Meanwhile, we found irregular margin features, homogeneous enhancement, and medium/low T2 signal intensity within the tumor associated with luminal subtypes. These findings are similar to other studies in the literature (23, 27). Due to the different intensities of tissue hyperplasia response, high-grade and fast-growing masses have a well-defined margin, while low-grade and slow-growing masses have a poorly defined margin and are spiculated, which may be explained by the desmoplastic reaction in adjacent breast tissues. This is the main reason for the detection of different morphological characteristics in different subtypes. What is striking is that morphological features such as round shape, circumscribed margin, and increased T2 signal intensity are also indicators of benign breast lesions (28). It should be kept in mind when evaluating breast MRI that these features are common in invasive breast cancer subtypes.

DCE-MRI has high sensitivity in assessing breast cancer, but there are differences in specificity. DWI can improve the diagnostic accuracy of DCE-MRI, and it is usually used as a component of multiparameter imaging to evaluate breast cancer (29–33). Some studies have reported the relationship between ADC values and prognostic factors in other subtypes of breast cancer, except TNBC (34, 35). Studies have reported that in luminal breast cancer, the average ADC value in the high proliferation group was significantly lower than that in the low proliferation group (36). However, few studies have reported the relationship between ADC value and the prognostic factors of breast cancer. In our study, the mean value of ADC was lower in the positive axillary lymph node, HER2-positive, ER-negative, and PR-negative groups. Although such difference was statistically significant only in the axillary lymph node status group, we assumed that the difference between ADC value and other prognostic factors may also be meaningful within a larger sample. A low ADC value is known as a hallmark of malignancy (28, 37). On this premise, we boldly hypothesized that ADC value might be a prognostic indicator of breast cancer.

As we all know, breast cancer is a heterogeneous disease, so early detection may be more helpful in clinical practice, such as early treatment planning and follow-up strategies. Unfortunately, the molecular typing of breast cancer can only be determined by the histopathological assessment of receptor status. Studies have shown that different molecular subtypes can be predicted by imaging findings, such as the shape of mass lesions, rim features, T2 signal intensity, and contrast enhancement features (23–26, 28–31). However, to our knowledge, there is no formal diagnostic method based on MRI.

Different from past research, we not only compared tumor lesions on MRI but also observed specific differences in the signal performance of surrounding tissues. The comparative analysis of paratumor signal intensity showed statistically significant differences among subtypes, which could help us better conduct molecular typing. Furthermore, all patients in our study received 3.0 T MRI. Compared to 1.5 T, our image resolution and quality were better, and these greatly enhanced the credibility of our study.

However, our study also had limitations. The biggest limitation was that the sample size was relatively small, with a limited number of some tumor subtypes. Secondly, this was a retrospective study and all the data were from a single institution, which may lead to selection bias. Finally, our study design did not collect patient prognostic data, which will be an important next step in evaluating the relationship between molecular subtypes and preoperative MRI.

## Conclusions

In summary, breast cancer subtypes, especially TNBC, exhibit multiple characteristic MRI features on DCE-MRI. With advances in imaging technology, the morphologic features of MRI can be used as imaging biomarkers to identify the molecular subtypes of breast cancer in the future. In addition, quantitative assessments of ADC values on DWI may also provide biological clues about molecular subtypes. Of course, a multicenter study with a larger sample size is needed to investigate this issue.

## Data availability statement

The original contributions presented in the study are included in the article/supplementary material. Further inquiries can be directed to the corresponding author.

## Author contributions

CW: conceptualization, methodology, and visualization. WL: supervision. JZ: data curation, writing—original draft preparation, and investigation. HC: writing—review and editing. All authors contributed to the article and approved the submitted version.

## Conflict of interest

The authors declare that the research was conducted in the absence of any commercial or financial relationships that could be construed as a potential conflict of interest.

## Publisher’s note

All claims expressed in this article are solely those of the authors and do not necessarily represent those of their affiliated organizations, or those of the publisher, the editors and the reviewers. Any product that may be evaluated in this article, or claim that may be made by its manufacturer, is not guaranteed or endorsed by the publisher.

## References

[1] Yamamoto Y Iwase H. Clinicopathological features and treatment strategy for triple-negative breast cancer. Int J Clin Oncol (2010) 15:341–51. doi: 10.1007/s10147-010-0106-1 20632057

[2] De Ronde JJ Hannemann J Halfwerk H Mulder L Straver ME Vrancken Peeters MJ. Concordance of clinical and molecular breast cancer subtyping in the context of preoperative chemotherapy response. Breast Cancer Res Treat (2010) 119(1):119–26. doi: 10.1007/s10549-009-0499-6 19669409

[3] Montagna E Bagnardi V Rotmensz N Viale G Cancello G Mazza M. Immunohistochemically defined subtypes and outcome in occult breast carcinoma with axillary presentation. Breast Cancer Res Treat (2011) 129(3):867–75. doi: 10.1007/s10549-011-1697-6 21822638

[4] Tran B Bedard PL. Luminal-b breast cancer and novel therapeutic targets. Breast Cancer Res (2011) 13(6):221. doi: 10.1186/bcr2904 22217398 PMC3326541

[5] Lam SW Jimenez CR Boven E. Breast cancer classification by proteomic technologies: current state of knowledge. Cancer Treat Rev (2014) 40(1):129–38. doi: 10.1016/j.ctrv.2013.06.006 23891266

[6] Li SP Padhani AR Taylor NJ Beresford MJ Ah-See ML Stirling JJ. Vascular characterisation of triple negative breast carcinomas using dynamic MRI. Eur Radiol (2011) 21(7):1364–73. doi: 10.1007/s00330-011-2061-2 21258931

[7] Dogan BE Gonzalez-Angulo AM Gilcrease M Dryden MJ Yang WT. Multimodality imaging of triple receptor-negative tumors with mammography, ultrasound, and MRI. AJR Am J Roentgenol (2010) 194(4):1160–6. doi: 10.2214/AJR.09.2355 20308526

[8] Mao C Jiang W Huang J Wang M Yan X Yang Z. Quantitative parameters of diffusion spectrum imaging: HER2 status prediction in patients with breast cancer. Front Oncol (2022) 12:817070. doi: 10.3389/fonc.2022.817070 35186753 PMC8850631

[9] Leithner D Bernard-Davila B Martinez DF Horvat JV Jochelson MS Marino MA. Radiomic signatures derived from diffusion-weighted imaging for the assessment of breast cancer receptor status and molecular subtypes. Mol Imaging Biol (2020) 22(2):453–61. doi: 10.1007/s11307-019-01383-w PMC7062654 31209778

[10] Podo F Buydens LM Degani H Hilhorst R Klipp E Gribbestad IS. Triple-negative breast cancer: present challenges and new perspectives. Mol Oncol (2010) 4(3):209–29. doi: 10.1016/j.molonc.2010.04.006 PMC5527939 20537966

[11] Youk JH Son EJ Chung J Kim JA Kim EK. Triple-negative invasive breast cancer on dynamic contrast-enhanced and diffusion-weighted MR imaging: comparison with other breast cancer subtypes. Eur Radiol (2012) 22(8):1724–34. doi: 10.1007/s00330-012-2425-2 22527371

[12] Horvat JV Bernard-Davila B Helbich TH Zhang M Morris EA Thakur SB. Diffusion-weighted imaging (DWI) with apparent diffusion coefficient (ADC) mapping as a quantitative imaging biomarker for prediction of immunohistochemical receptor status, proliferation rate, and molecular subtypes of breast cancer. J Magn Reson Imaging (2019) 50(3):836–46. doi: 10.1002/jmri.26697 PMC6767396 30811717

[13] Martincich L Deantoni V Bertotto I Redana S Kubatzki F Sarotto I. Correlations between diffusion-weighted imaging and breast cancer biomarkers. Eur Radiol (2012) 22(7):1519–28. doi: 10.1007/s00330-012-2403-8 22411304

[14] American College of Radiology. Breast Imaging Reporting and Data System (BI-RADS). 5rd ed. Reston Va: American College of Radiology (2013).

[15] Li J Chen Z Su K Zeng J. Clinicopathological classification and traditional prognostic indicators of breast cancer. Int J Clin Exp Pathol (2015) 8(7):8500–5. PMC4555752 26339424

[16] Goldhirsch A Winer EP Coates AS Gelber RD Piccart-Gebhart M Thürlimann B. Personalizing the treatment of women with early breast cancer: highlights of the St gallen international expert consensus on the primary therapy of early breast cancer 2013. Ann Oncol (2013) 24(9):2206–23. doi: 10.1093/annonc/mdt303 PMC3755334 23917950

[17] Theriault RL Carlson RW Allred C Anderson BO Burstein HJ Edge SB. National comprehensive cancer network. breast cancer, version 3.2013: featured updates to the NCCN guidelines. J Natl Compr Canc Netw (2013) 11(7):753–60. doi: 10.6004/jnccn.2013.0098 PMC3991132 23847214

[18] Giuliano AE Hunt KK Ballman KV Beitsch PD Whitworth PW Blumencranz PW. Axillary dissection vs no axillary dissection in women with invasive breast cancer and sentinel node metastasis: a randomized clinical trial. JAMA (2011) 305(6):569–75. doi: 10.1001/jama.2011.90 PMC5389857 21304082

[19] Li Y Moran MS Huo Q Yang Q Haffty BG. Post-mastectomy radiotherapy for breast cancer patients with t1-t2 and 1-3 positive lymph nodes: a meta-analysis. PLoS One (2013) 8(12):e81765. doi: 10.1371/journal.pone.0081765 24312582 PMC3849378

[20] Kim JJ Kim JY Suh HB Hwangbo L Lee NK Kim S. Characterization of breast cancer subtypes based on quantitative assessment of intratumoral heterogeneity using dynamic contrast-enhanced and diffusion-weighted magnetic resonance imaging. Eur Radiol (2022) 32(2):822–33. doi: 10.1007/s00330-021-08166-4 34345946

[21] Wolff AC Hammond ME Hicks DG Dowsett M McShane LM Allison KH. Recommendations for human epidermal growth factor receptor 2 testing in breast cancer: American society of clinical Oncology/College of American pathologists clinical practice guideline update. J Clin Oncol (2013) 31(31):3997–4013. doi: 10.1200/JCO.2013.50.9984 24101045

[22] Grimm LJ Johnson KS Marcom PK Baker JA Soo MS. Can breast cancer molecular subtype help to select patients for preoperative MR imaging? Radiology (2015) 274(2):352–8. doi: 10.1148/radiol.14140594 25325325

[23] Sung JS Jochelson MS Brennan S Joo S Wen YH Moskowitz C. MR imaging features of triple-negative breast cancers. Breast J (2013) 19(6):643–9. doi: 10.1111/tbj.12182 24015869

[24] Costantini M Belli P Distefano D Bufi E Matteo MD Rinaldi P. Magnetic resonance imaging features in triple-negative breast cancer: comparison with luminal and HER2-overexpressing tumors. Clin Breast Cancer (2012) 12(5):331–9. doi: 10.1016/j.clbc.2012.07.002 23040001

[25] Angelini G Marini C Iacconi C Mazzotta D Moretti M Picano E. Magnetic resonance (MR) features in triple negative breast cancer (TNBC) vs receptor positive cancer (nTNBC). Clin Imaging (2018) 49:12–6. doi: 10.1016/j.clinimag.2017.10.016 29120811

[26] Öztürk VS Polat YD Soyder A Tanyeri A Karaman CZ Taşkın F. The relationship between MRI findings and molecular subtypes in women with breast cancer. Curr Probl Diagn Radiol (2020) 49(6):417–21. doi: 10.1067/j.cpradiol.2019.07.003 31351695

[27] Navarro Vilar L Alandete Germán SP Medina García R Blanc García E Camarasa Lillo N Vilar Samper J. MR imaging findings in molecular subtypes of breast cancer according to BIRADS system. Breast J (2017) 23(4):421–8. doi: 10.1111/tbj.12756 28067435

[28] Tezcan S Ozturk FU Uslu N Akcay EY. The role of combined diffusion-weighted imaging and dynamic contrast-enhanced MRI for differentiating malignant from benign breast lesions presenting washout curve. Can Assoc Radiol J (2021) 72(3):460–9. doi: 10.1177/0846537120907098 32157892

[29] Xie T Zhao Q Fu C Bai Q Zhou X Li L. Differentiation of triple-negative breast cancer from other subtypes through whole-tumor histogram analysis on multiparametric MR imaging. Eur Radiol (2019) 29(5):2535–44. doi: 10.1007/s00330-018-5804-5 30402704

[30] Montemezzi S Camera L Giri MG Pozzetto A Caliò A Meliadò G. Is there a correlation between 3T multiparametric MRI and molecular subtypes of breast cancer? Eur J Radiol (2018) 108:120–7. doi: 10.1016/j.ejrad.2018.09.024 30396643

[31] Suo S Cheng F Cao M Kang J Wang M Hua J. Multiparametric diffusion-weighted imaging in breast lesions: Association with pathologic diagnosis and prognostic factors. J Magn Reson Imaging (2017) 46(3):740–50. doi: 10.1002/jmri.25612 28139036

[32] Horvat JV Iyer A Morris EA Apte A Bernard-Davila B Martinez DF. Histogram analysis and visual heterogeneity of diffusion-weighted imaging with apparent diffusion coefficient mapping in the prediction of molecular subtypes of invasive breast cancers. Contrast Media Mol Imaging (2019) 2019:2972189. doi: 10.1155/2019/2972189 31819738 PMC6893252

[33] Park SH Choi HY Hahn SY. Correlations between apparent diffusion coefficient values of invasive ductal carcinoma and pathologic factors on diffusion-weighted MRI at 3.0 Tesla. J Magn Reson Imaging (2015) 41(1):175–82. doi: 10.1002/jmri.24519 24353241

[34] Choi SY Chang YW Park HJ Kim HJ Hong SS Seo DY. Correlation of the apparent diffusion coefficiency values on diffusion-weighted imaging with prognostic factors for breast cancer. Br J Radiol (2012) 85(1016):e474–9. doi: 10.1259/bjr/79381464 PMC3587081 22128125

[35] Kim SY Kim EK Moon HJ Yoon JH Koo JS Kim SG. Association among T2 signal intensity, necrosis, ADC and ki-67 in estrogen receptor-positive and HER2-negative invasive ductal carcinoma. Magn Reson Imaging (2018) 54:176–82. doi: 10.1016/j.mri.2018.08.017 PMC7383359 30172938

[36] Mori N Ota H Mugikura S Takasawa C Ishida T Watanabe G. Luminal-type breast cancer: correlation of apparent diffusion coefficients with the ki-67 labeling index. Radiology (2015) 274(1):66–73. doi: 10.1148/radiol.14140283 25203132

[37] Che SN Li J Xue M Song Y Zhao LY Guo N. The value of synthetic MRI in differential diagnosis of benign and malignant breast lesions. Zhonghua Zhong Liu Za Zhi (2021) 43(8):872–7. doi: 10.3760/cma.j.cn112152-20210322-00254 34407594

